# Randomized, double-blind, placebo-controlled study to evaluate the efficacy and safety of etanercept in patients with moderately active rheumatoid arthritis despite DMARD therapy

**DOI:** 10.1186/s40064-015-0895-9

**Published:** 2015-03-05

**Authors:** Kathryn Hobbs, Atul Deodhar, Brian Wang, Bojena Bitman, Joyce Nussbaum, James Chung, David H Collier

**Affiliations:** Denver Arthritis Clinic, 200 Spruce Street, Suite 100, Denver, CO 80230 USA; Oregon Health & Science University, 3181 SW Sam Jackson Park Road, Portland, OR 97239 USA; Amgen Inc, 1120 Veterans Boulevard, South San Francisco, CA 94080 USA; Amgen Inc, One Amgen Center Drive, Thousand Oaks, CA 91320 USA

**Keywords:** Rheumatoid arthritis, Etanercept, Randomized controlled trial

## Abstract

This study evaluated the efficacy and safety of adding etanercept to disease-modifying antirheumatic drugs (DMARDs) in patients with moderately active rheumatoid arthritis (RA). This randomized, double-blind, placebo-controlled study (ClinicalTrials.gov #NCT01313208) enrolled RA patients with Disease Activity Score using 28 joints with C-reactive protein (DAS28-CRP) >3.2 and ≤5.1 (moderate disease) despite stable DMARD therapy. Patients were randomized to etanercept 50 mg or placebo weekly for 12 weeks; all patients then received etanercept 50 mg weekly through week 24. Primary endpoint was low disease activity (LDA) at week 12; secondary endpoints included DAS28-CRP remission at week 12; Clinical Disease Activity Index (CDAI) and Simplified Disease Activity Index (SDAI) LDA; American College of Rheumatology (ACR) responses; change in Health Assessment Questionnaire Disability Index (HAQ-DI), and safety. For 210 patients with moderate disease at screening, (104 placebo; 106 etanercept), only 58% still had moderate disease at baseline. At week 12, 33% on etanercept and 21% on placebo achieved LDA (*P* = 0.055); remission was achieved in 19% and 12%, respectively (*P* = 0.14). At week 12, ACR20, ACR50, and ACR70 responses were observed in 29%, 13%, and 1% respectively, in patients on placebo, and 41%, 21%, and 6% of patients on etanercept. Mean (SD) change from baseline in HAQ-DI score was −0.20 (0.43) for placebo patients and −0.39 (0.54) for etanercept patients at week 12. No new safety signals were observed. LDA was achieved by more patients on etanercept than placebo in patients with moderate disease at screening, but the difference was not statistically significant at week 12.

## Introduction

The American College of Rheumatology (ACR) recommends a treatment goal of either low disease activity (LDA) or remission in all patients with early rheumatoid arthritis (RA) and established RA by using disease-modifying antirheumatic drugs (DMARDs) or biologic agents (Singh et al. 2012). Etanercept, a modified p75 receptor of tumor necrosis factor (TNF) that inhibits the action of TNF, has been shown to be efficacious for the treatment of moderate to severe RA in patients with early (Bathon et al. 2000) and established (Moreland et al. 2006) disease. Subgroup analyses have indicated that patients with moderately active disease may be more likely to achieve better disease status (LDA or remission) with etanercept treatment than patients with more severe disease, despite smaller absolute improvements in disease severity (Keystone et al. 2009). The original trials did not stratify by disease activity and the subgroup of patients with moderate disease activity was relatively small. A prospective trial would better characterize the efficacy profile of etanercept in patients with moderately active disease, a medically important subset of RA patients.

The objective of this study was to evaluate whether adding etanercept 50 mg per week to standard-of-care DMARD therapy in patients with moderately active RA is superior in inducing very good control of disease compared with continued DMARD therapy.

## Methods

### Study design

This was a phase 4, prospective, randomized, double-blind, placebo-controlled study. After completing all assessments during the screening window of up to 31 days, patients were randomized (1:1) to receive etanercept 50 mg weekly or placebo administered subcutaneously for 12 weeks. After week 12, all patients received etanercept 50 mg weekly for an additional 12 weeks. Patients were followed for an additional 4 weeks following the last dose of investigational product to monitor safety. Randomization was accomplished using an Interactive Voice Response System. Assignment to treatment arm was based on a computer-generated randomization schedule that was prepared by the sponsor before the start of the study, and used randomly permuted blocks. Randomization was stratified by use of methotrexate at baseline. Patients, site personnel, and investigators were blinded to treatment assignment.

### Patients

Eligible patients were ≥ 18 and ≤ 80 years of age at screening and had a diagnosis of RA per the 1987 ACR classification criteria (Arnett et al. 1988) for ≥ 6 months before screening. Patients were required to have moderately active disease as defined by a Disease Activity Score based on 28 joints with C-reactive protein as the indicator of inflammation (DAS28-CRP) > 3.2 and ≤ 5.1 (Fransen and van Riel 2005) and ≥ 3 swollen joints and ≥ 3 tender joints. CRP levels were measured using a central laboratory. Samples for CRP testing were collected at screening (for investigators to identify patients with moderately active disease based on DAS28-CRP calculations) and at baseline. Several days elapsed between collection of samples and availability of CRP results, so baseline DAS28-CRP was calculated retrospectively and randomization at baseline was based solely on swollen/tender joints at the screening visit. Patients had to be taking methotrexate for ≥ 12 weeks with a stable dose of 15–25 mg weekly for ≥ 8 weeks prior to baseline (lower doses were allowed at the investigator’s discretion); patients with contraindications to methotrexate were allowed to enroll if they were using sulfasalazine, leflunomide, minocycline, and/or hydroxychloroquine. Exclusion criteria included: prosthetic joint infection within 5 years or native joint infection within 1 year of screening, Class IV RA according to ACR revised criteria (Hochberg et al. 1992), diagnosis of Felty’s syndrome, use of > 1 commercially available or experimental biologic DMARD (use of 1 biologic DMARD was allowed if the patient had received no more than 8 weeks of treatment and did not use the DMARD within 2 months of the first dose in this study), serious infection requiring hospitalization or intravenous antibiotics within 8 weeks before screening, active infection requiring anti-infectives within 28 days prior to first dose, significant concurrent medical conditions, or laboratory abnormalities at screening.

### Study endpoints

Endpoints included the proportion of patients with DAS28-CRP LDA (DAS28-CRP < 3.2; primary endpoint) and remission (DAS28-CRP < 2.6; key secondary endpoint) (Aletaha and Smolen 2005) at week 12. Additional secondary endpoints included rates of Clinical Disease Activity Index (CDAI) LDA (score ≤ 10) and remission (score ≤ 2.8) (Aletaha and Smolen 2005); rates of Simplified Disease Activity Index (SDAI) LDA (score ≤ 11) and remission (score ≤ 3.3) (Aletaha and Smolen 2005); rates of 20%, 50%, and 70% improvement in ACR criteria (ACR20, ACR50, and ACR70) (Felson et al. 1995); changes in Health Assessment Questionnaire Disability Index (HAQ-DI) (Wolfe et al. 1988); and safety. Safety endpoints included the nature, frequency, severity and relationship to treatment of all adverse events (AEs). Efficacy and patient-reported outcomes were assessed at baseline and weeks 2, 4, 8, 12, 16, 20, and 24.

### Statistical considerations

The hypothesis tested was that adding etanercept in RA patients with moderately active disease despite DMARD therapy would yield a greater proportion of patients with LDA and remission than continued DMARD therapy only, as measured by DAS28-CRP at week 12. A sample size of 100 patients per treatment arm was estimated to provide 83% power to detect a difference in the proportion of patients achieving DAS28-CRP LDA with alpha of 0.05.

The primary efficacy endpoint was compared between the two treatment arms using the Mantel-Haenszel test stratified by methotrexate use (yes or no) at baseline. DAS28-CRP was also summarized as a continuous variable by treatment group. Last observation carried forward was used to impute missing data for the primary analysis for patients without an assessment at week 12. Primary and secondary endpoints were tested sequentially, i.e. secondary endpoints were tested only if the primary endpoint was statistically significant.

All efficacy endpoints were evaluated using the primary analysis set, which comprised all randomized patients. Additional efficacy analyses were performed on the subset of patients who had moderate disease at baseline. Safety endpoints were analyzed using the safety analysis set, which included all randomized patients who received at least one dose of any investigational product, and were analyzed based on treatment received. AEs were summarized and coded using the Medical Dictionary for Regulatory Activities (MedDRA) version 15.1. Statistical analyses were performed using SAS version 9.2 (SAS Institute Inc., Cary, NC, USA).

### Ethical standards

This study was conducted in accordance with the Helsinki declaration. The study protocol and consent were approved by the institutional review board at each study site. All patients provided signed informed consent prior to initiation of any study-related procedures.

## Results

### Patients

**Figure 1:**
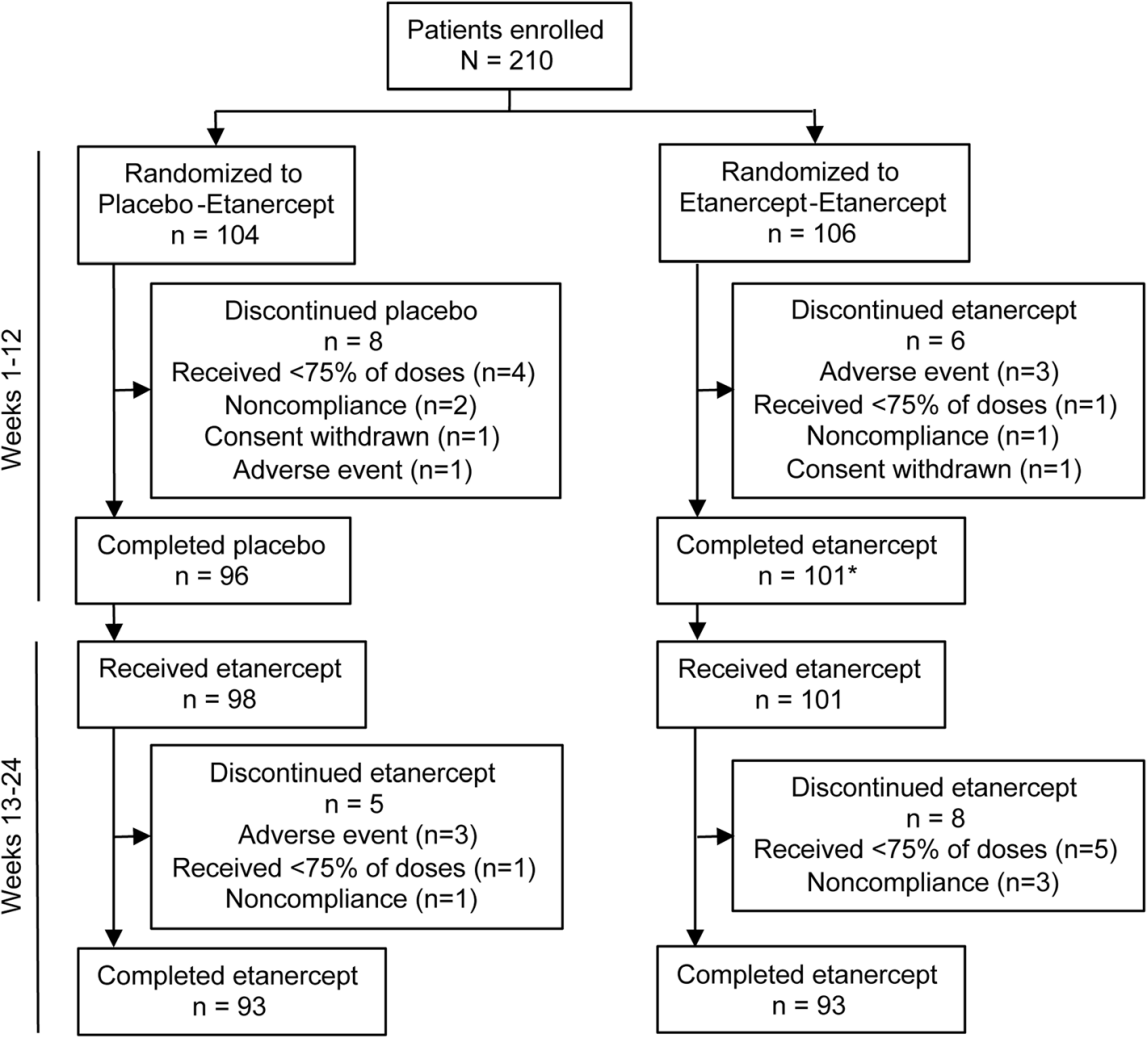
**Patient disposition.** *One patient was counted as having both completed etanercept and discontinuing etanercept. The patient received all 12 doses of etanercept during weeks 1 through 12 of the study, but ended treatment on the day of the last dose because of an adverse event that required protocol-prohibited treatments.

A total of 210 patients were enrolled in the study from 38 centers in the US and Canada; 104 patients were randomized to the placebo-etanercept group and 106 were randomized to the etanercept-etanercept group. The study was conducted from March 31, 2011 (first patient enrolled) through May 22, 2013 (last patient visit). Most patients were women (77%), most were white (86%), the mean (standard deviation [SD]) age was 56.0 (12.4) years, and the mean (SD) duration of RA was 7.8 (9.8) years (Table 1). Patients were required to have moderate disease activity at screening to qualify for enrollment in the study; however, at baseline only 122 patients (58%) had moderate disease when administration of study drugs was initiated. The screening period ranged up to 35 days for most patients, with a median of 15 days. Of the 88 patients who no longer had moderate disease activity at baseline, 3 had improved to LDA and 85 had worsened. During the double-blind portion of the trial (weeks 1–12), 6.7% of patients discontinued treatment, and during weeks 13–24, 6.2% of patients discontinued treatment (Figure 1). All 210 patients were included in the primary analysis set and the safety analysis set.

**Table 1:** **Demographic and clinical characteristics at screening (primary analysis set)**

	**Placebo-etanercept (N = 104)**	**Etanercept-etanercept (N = 106)**	**All patients (N = 210)**
Age, mean years (SD)	55.5 (12.8)	56.5 (12.1)	56.0 (12.4)
Sex, n women (%)	86 (82.7)	75 (70.8)	161 (76.7)
Race, n (%)			
White	90 (86.5)	91 (85.8)	181 (86.2)
Black/African American	8 (7.7)	9 (8.5)	17 (8.1)
Asian	3 (2.9)	2 (1.9)	5 (2.4)
Other	3 (2.9)	4 (3.8)	7 (3.3)
BMI, mean kg/m^2^ (SD)	29.3 (6.6)	30.6 (7.7)	30.0 (7.2)
Duration of RA, mean years (SD)	7.4 (8.1)	8.3 (11.2)	7.8 (9.8)
DAS28-CRP, mean (SD)	4.9 (0.8)	4.9 (0.7)	4.9 (0.8)

*SD*: standard deviation; *BMI*: body mass index; *RA*: rheumatoid arthritis; *DAS28-CRP*: Disease Activity Score based on 28 joints with C-reactive protein.

### Efficacy outcomes

**Figure 2:**
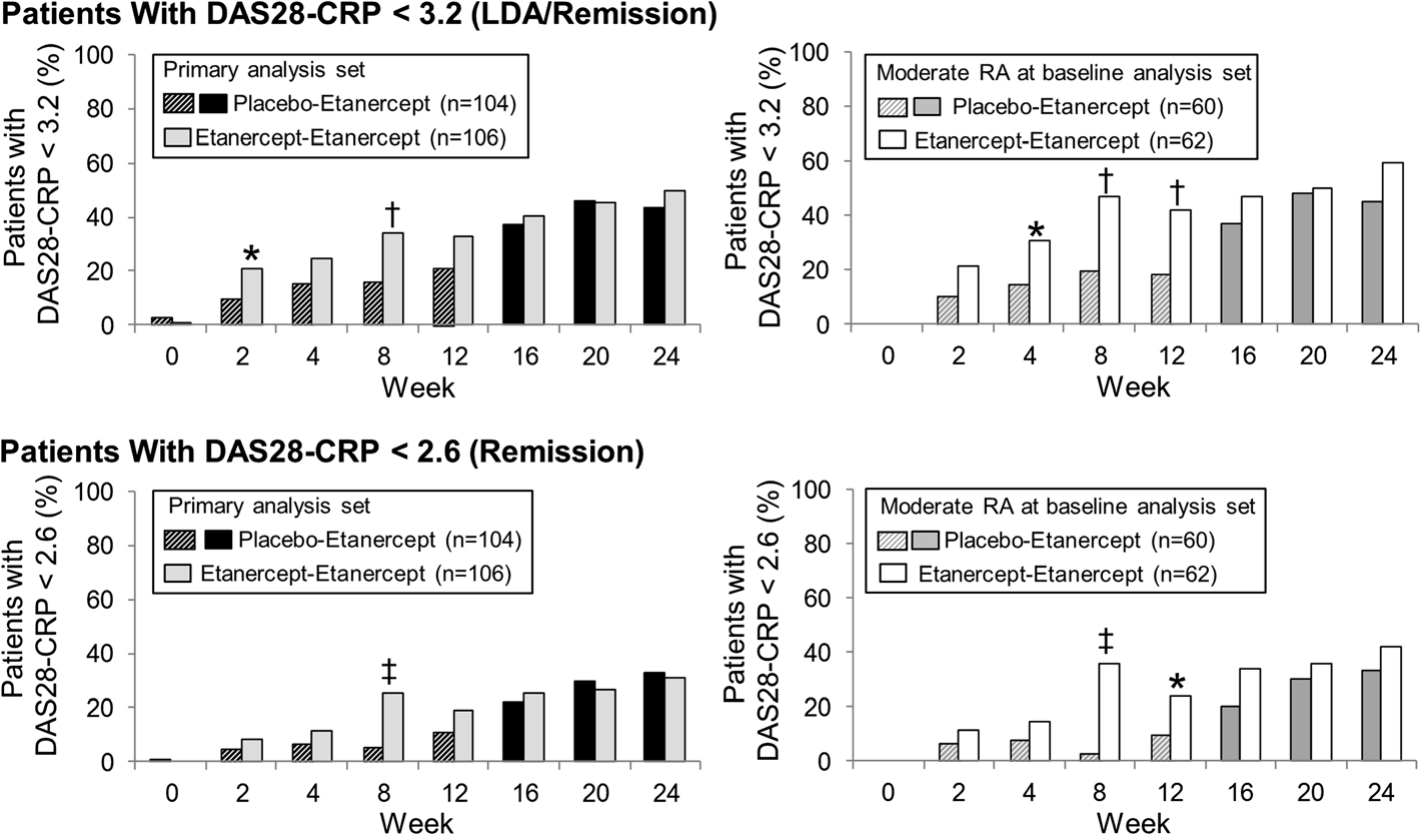
**Rates of DAS28-CRP LDA and remission.** The percentages of patients with DAS28-CRP < 3.2 (LDA; top panels) and < 2.6 (remission; bottom panels) are shown. Data are shown for the primary analysis set (left panels) and the subset of patients with moderate RA at baseline (right panels). Patients in the placebo-etanercept group (black bars in left panels; gray bars in right panels) received placebo (hashed bars) in the first 12 weeks and etanercept through week 24 and patients in the etanercept-etanercept group received etanercept (gray bars in left panels; white bars in right panels) throughout the study. *P < 0.05; ^†^ *P* < 0.01; ^‡^ *P* < 0.001 for comparison between groups. DAS28-CRP: Disease Activity Score based on 28 joints with C-reactive protein; LDA: low disease activity; RA: rheumatoid arthritis.

**Figure 3:**
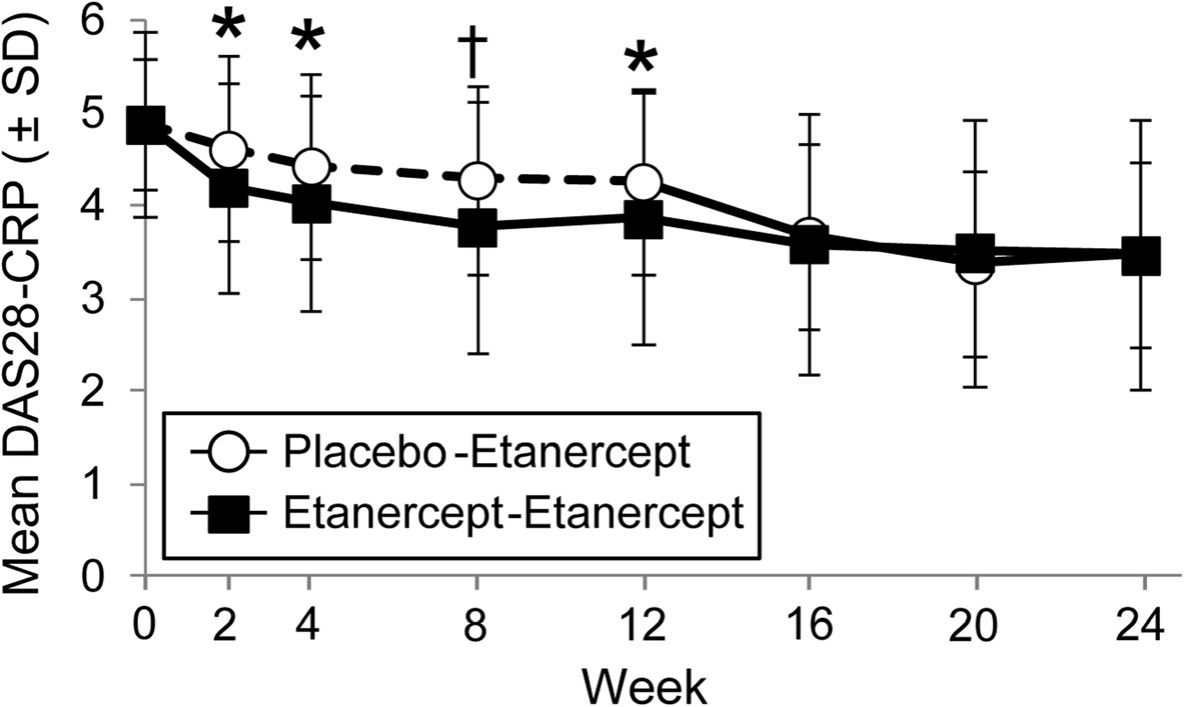
**DAS28-CRP values.** Mean DAS28-CRP values are shown for the placebo-etanercept (circles) and etanercept-etanercept (squares) groups. Dotted lines indicate the period when the placebo-etanercept group received placebo. Error bars represent standard deviations. *P < 0.05; ^†^ *P* < 0.01 for comparison between groups. DAS28-CRP: Disease Activity Score based on 28 joints with C-reactive protein; SD, standard deviation.

The study failed to meet the primary endpoint. At week 12, the percentage of patients who achieved LDA was not significantly different between the placebo-etanercept group (21%) and the etanercept-etanercept group (33%; *P* = 0.055). Rates of DAS28-CRP LDA were statistically significantly different at week 8 (placebo-etanercept group 16%, etanercept-etanercept group 34%; nominal *P* = 0.003) (Figure 2); however, improvement in DAS28-CRP in the placebo-etanercept group and lack of continued improvement in the etanercept-etanercept group was observed between weeks 8 and 12 (Figure 3). In a post hoc analysis of patients who had moderate disease at baseline, 18% of patients in the placebo-etanercept group and 42% of patients in the etanercept-etanercept group had achieved DAS28-CRP LDA at week 12 (*P* = 0.005) (Figure 2).

Because the primary endpoint did not reach statistical significance, formal testing of secondary endpoints was not performed. Key secondary endpoints of CDAI and SDAI rates of LDA and remission and ACR responses are shown in Table 2. Similar to results of the primary endpoint, differences between treatment groups were greatest at week 8 for all key secondary endpoints.

**Table 2:** **Key secondary endpoints: rates of CDAI and SDAI LDA and remission, ACR responses, improvements in HAQ-DI (primary analysis set; LOCF imputation)**

	**Placebo-etanercept (N = 104)**	**Etanercept-etanercept (N = 106)**
CDAI LDA (score ≤10), n (%)		
Week 2	9 (9.1)	13 (12.3)
Week 8	16 (15.4)	31 (29.2)
Week 12	22 (21.2)	27 (25.5)
Week 24	43 (41.3)	49 (46.2)
CDAI remission (score ≤2.8), n (%)		
Week 2	1 (1.0)	2 (1.9)
Week 8	0 (0.0)	3 (2.8)
Week 12	1 (1.0)	4 (3.8)
Week 24	4 (3.8)	9 (8.5)
SDAI LDA (score ≤11), n (%)		
Week 2	8 (8.2)	13 (12.4)
Week 8	15 (14.4)	32 (30.2)
Week 12	22 (21.2)	25 (23.6)
Week 24	41 (39.4)	48 (45.3)
SDAI remission (score ≤3.3), n (%)		
Week 2	1 (1.0)	1 (1.0)
Week 8	0 (0.0)	6 (5.7)
Week 12	2 (1.9)	6 (5.7)
Week 24	7 (6.7)	11 (10.4)
ACR20 response, n (%)		
Week 2	15 (14.9)	30 (28.6)
Week 8	24 (23.1)	53 (50.0)
Week 12	30 (28.8)	43 (40.6)
Week 24	48 (46.2)	53 (50.0)
ACR50 response, n (%)		
Week 2	3 (3.0)	6 (5.7)
Week 8	5 (4.8)	21 (19.8)
Week 12	13 (12.5)	22 (20.8)
Week 24	30 (28.8)	35 (33.0)
ACR70 response, n (%)		
Week 2	0 (0.0)	2 (1.9)
Week 8	0 (0.0)	6 (5.7)
Week 12	1 (1.0)	6 (5.7)
Week 24	13 (12.5)	17 (16.0)
Change from baseline in HAQ-DI, mean score change (SD)		
Week 2	−0.09 (0.39)	−0.27 (0.43)
Week 8	−0.21 (0.42)	−0.37 (0.47)
Week 12	−0.20 (0.43)	−0.39 (0.54)
Week 24	−0.45 (0.52)	−0.48 (0.58)

*CDAI*: Clinical Disease Activity Index; *SDAI*: Simplified Disease Activity Index; *LDA*: low disease activity; *ACR*: American College of Rheumatology; *HAQ-DI*: Health Assessment Questionnaire Disability Index; *LOCF*: last observation carried forward; *SD*: standard deviation.

### Patient-reported outcomes

Patients in the etanercept-etanercept group showed improvements from baseline in HAQ-DI score throughout the study (Table 2). Patients in the placebo-etanercept group showed minimal improvements from baseline through week 12, but at week 24 had similar improvements from baseline as patients in the etanercept-etanercept group.

### Safety results

During the 12-week double-blind portion of the study, 61% of patients in the placebo-etanercept group and 67% of the etanercept-etanercept group reported an AE (Table 3). The most commonly reported AEs through week 12 included injection site erythema (1.0% placebo-etanercept; 11.3% etanercept-etanercept), headache (10.6%; 7.5%), injection site pruritus (1.9%; 6.6%), and injection site rash (1.0%; 6.6%). Overall, 78% of all patients reported an AE through 24 weeks of treatment. The most commonly reported AEs through week 24 included injection site erythema (10.6% placebo-etanercept; 12.3% etanercept-etanercept), RA worsening/flare (11.5%; 11.3%), headache (15.4%; 8.5%), and upper respiratory tract infection (12.5%; 7.5%). Serious AEs were reported in 7 patients (3 patients in the placebo-etanercept group and 4 patients in the etanercept-etanercept group) through 24 weeks of treatment. No opportunistic infections, malignancies, or deaths were reported during the study. No new or unexpected safety signals were observed.

**Table 3:** **Summary of safety (safety analysis set)**

	**Placebo-etanercept (N = 104)**	**Etanercept-etanercept (N = 106)**	**All patients (N = 210)**
Patients reporting an AE, n (%)			
Weeks 1–12 (double-blind portion)	63 (60.6)	71 (67.0)	134 (63.8)
Weeks 1–24	80 (76.9)	83 (78.3)	163 (77.6)
Patients reporting an SAE, n (%)			
Weeks 1–12 (double-blind portion)	2 (1.9)	3 (2.8)	5 (2.4)
Weeks 1–24	3 (2.9)	4 (3.8)	7 (3.3)
Patients reporting an SIE, n (%)			
Weeks 1–12 (double-blind portion)	0 (0)	0 (0)	0 (0)
Weeks 1–24	0 (0)	1 (0.9)	1 (0.5)
Patients reporting an infection, n (%)			
Weeks 1–12 (double-blind portion)	23 (22.1)	30 (28.3)	53 (25.2)
Weeks 1–24	46 (44.2)	39 (36.8)	85 (40.5)

*AE*: adverse event; *SAE*: serious adverse event; *SIE*: serious infectious event.

## Discussion

Patients with moderate disease activity despite treatment with DMARDs represent a medically important subset of patients with RA, as their first-line therapy has failed to achieve or sustain LDA or remission. As these patients have been shown to achieve better clinical responses to etanercept therapy than patients with severe disease (Keystone et al. 2009), this study therefore was designed to further investigate the efficacy and safety of adding etanercept to methotrexate in this patient population. Although the primary endpoint of the study was not reached for the entire study population, a post hoc analysis of the subset of patients who fulfilled the entry criterion of moderate disease activity at baseline showed that the addition of etanercept to methotrexate resulted in a greater proportion of patients achieving DAS28-CRP LDA at week 12.

Several possible reasons for the failure of the study to meet its primary endpoint have been identified. There was a protocol failure with respect to how patients with moderate disease were screened and enrolled. The protocol required the site to calculate the DAS28-CRP and confirm moderate disease only at screening but should have required this assessment at both screening and at baseline prior to enrollment. A solution to this issue could have been the use of erythrocyte sedimentation rate (ESR), which can be performed locally. Measuring the level of inflammation with ESR instead of CRP would have avoided the time delay in measuring the level of inflammation for calculating disease severity. Another potential solution would have been to require that moderate disease activity was stable over a specific period, such as 3 months, before enrollment in the study. Moderate disease activity may represent a transient state, with some patients rapidly worsening to severe disease or improving to LDA. Additionally, patients were allowed to receive nonbiologic DMARDs at study entry; DMARDs initiated within 3 months of enrollment into this study may not have reached their full effect when patients were evaluated during the screening window. Use of corticosteroids did not appear to influence the study results (data not shown). Finally, the measurement of disease activity was based on a composite global measurement and may not have been sensitive enough to provide a clear distinction between moderate and high disease activity.

The primary limitation of the study was the insufficient number of patients with moderate disease at baseline based on the power analysis requirement to determine the minimum number of patients required to test the hypothesis. Surprisingly, rates of DAS28-CRP LDA were statistically significant between etanercept and placebo at week 8, but failed to reach significance at week 12. Between weeks 8 and 12 of this study, unexpected improvements in DAS28-CRP in the placebo-etanercept group and lack of continued improvements in the etanercept-etanercept group were observed. All lots of drugs dispensed in the study were scrutinized, and no discrepancies were found. Rates of ACR responses at week 12 reflected differences in response between the patients in this trial and those enrolled in prior etanercept trials. In the pivotal trial of etanercept plus methotrexate combination therapy (Weinblatt et al. 1999), 66% of patients on combination therapy achieved an ACR20 response at week 12 compared with only 41% of patients in our study. Notably, ACR20 response rates at week 12 were similar in patients receiving placebo plus methotrexate in the pivotal trial (33%) and our study (29%).

The results of this study revealed a trend toward better clinical outcomes in patients on etanercept plus methotrexate therapy compared with methotrexate alone. Within the spectrum of moderately active disease, changes in status (ie, moderate to severe disease) can be frequent and disease activity scores at any given time may not accurately reflect these changes. Protocols that do not confirm the hypothesis such as this may offer results that are clinically significant but difficult to reconcile. However, they also provide information for improving designs of future studies.
